# Agreement between continuous cardiac output measured by the fourth-generation FloTrac/Vigileo system and a pulmonary artery catheter in adult liver transplantation

**DOI:** 10.1038/s41598-022-14988-z

**Published:** 2022-07-01

**Authors:** Yutaka Murata, Takumi Imai, Chikashi Takeda, Toshiyuki Mizota, Shuji Kawamoto

**Affiliations:** 1 Department of Anesthesia, Kyoto University Hospital, 54 Shogoin Kawahara-cho, Sakyo-ku, Kyoto, 606-8507 Japan; 2 Department of Medical Statistics, Osaka Metropolitan University, 1-4-3 Asahimachi, Abeno-ku, Osaka, 545-8585 Japan

**Keywords:** Blood flow, Data processing

## Abstract

In liver transplantation for end-stage liver failure, monitoring of continuous cardiac output (CCO) is used for circulatory management due to hemodynamic instability. CCO is often measured using the minimally invasive FloTrac/Vigileo system (FVS-CCO), instead of a highly invasive pulmonary artery catheter (PAC-CCO). The FVS has improved accuracy due to an updated cardiac output algorithm, but the effect of this change on the accuracy of FVS-CCO in liver transplantation is unclear. In this study, we assessed agreement between fourth-generation FVS-CCO and PAC-CCO in 20 patients aged ≥ 20 years who underwent scheduled or emergency liver transplantation at Kyoto University Hospital from September 2019 to June 2021. Consent was obtained before surgery and data were recorded throughout the surgical period. Pearson correlation coefficient (*r*), Bland–Altman and 4-quadrant plot analyses were performed on the extracted data. A total of 1517 PAC-CCO vs. FVS-CCO data pairs were obtained. The mean PAC-CCO was 8.73 L/min and the mean systemic vascular resistance was 617.5 dyne·s·cm^-5^, *r* was 0.48, bias was 1.62 L/min, the 95% limits of agreement were − 3.04 to 6.27, and the percentage error was 54.36%. These results show that agreement and trending between fourth-generation FVS-CCO and PAC-CCO are low in adult liver transplant recipients.

## Introduction

Liver transplantation in patients with end-stage liver failure is a challenging procedure for anesthesiologists because of hemodynamic instability (referred to as a hyperdynamic state), intermittent obstruction of venous return, graft reperfusion and post-reperfusion syndrome, and significant blood loss^1^. Continuous cardiac output (CCO) monitoring is useful in this surgery. Intermittent cardiac output monitoring with a pulmonary artery catheter (PAC-ICO) is currently used clinically, and CCO monitoring with a thermodilution pulmonary artery catheter (PAC-CCO) is also used and correlates well with PAC-ICO^2^. However, use of a PAC has been questioned due to its high invasiveness and the risk of fatal complications such as complete atrioventricular block, right ventricular perforation, pulmonary artery perforation, infectious endocarditis, and pulmonary embolism^3^.

CCO monitored by the minimally invasive FloTrac/Vigileo system (FVS-CCO) has been used recently as an alternative to PAC-ICO and PAC-CCO. The FVS measures cardiac output (CO) by connecting to the radial arterial line and analyzing arterial pressure waveforms. The accuracy of measurement has improved due to use of an updated CO algorithm. The third-generation algorithm (ver. 3.02, 2009) had improved accuracy in a hyperdynamic state^4^, and the latest fourth-generation algorithm (ver. 4.00, 2014) adjusts for acute SVR changes using a new correction factor with a short adaptation interval^5^. However, it is unclear if these changes improve the accuracy of FVS-CCO in liver transplantation. Therefore, in this study, we assessed the agreement of fourth-generation FVS-CCO, which is more accurate in a hyperdynamic state and with administration of vasoconstrictors, with PAC-CCO in adult liver transplant recipients.

## Results

**Figure 1:**
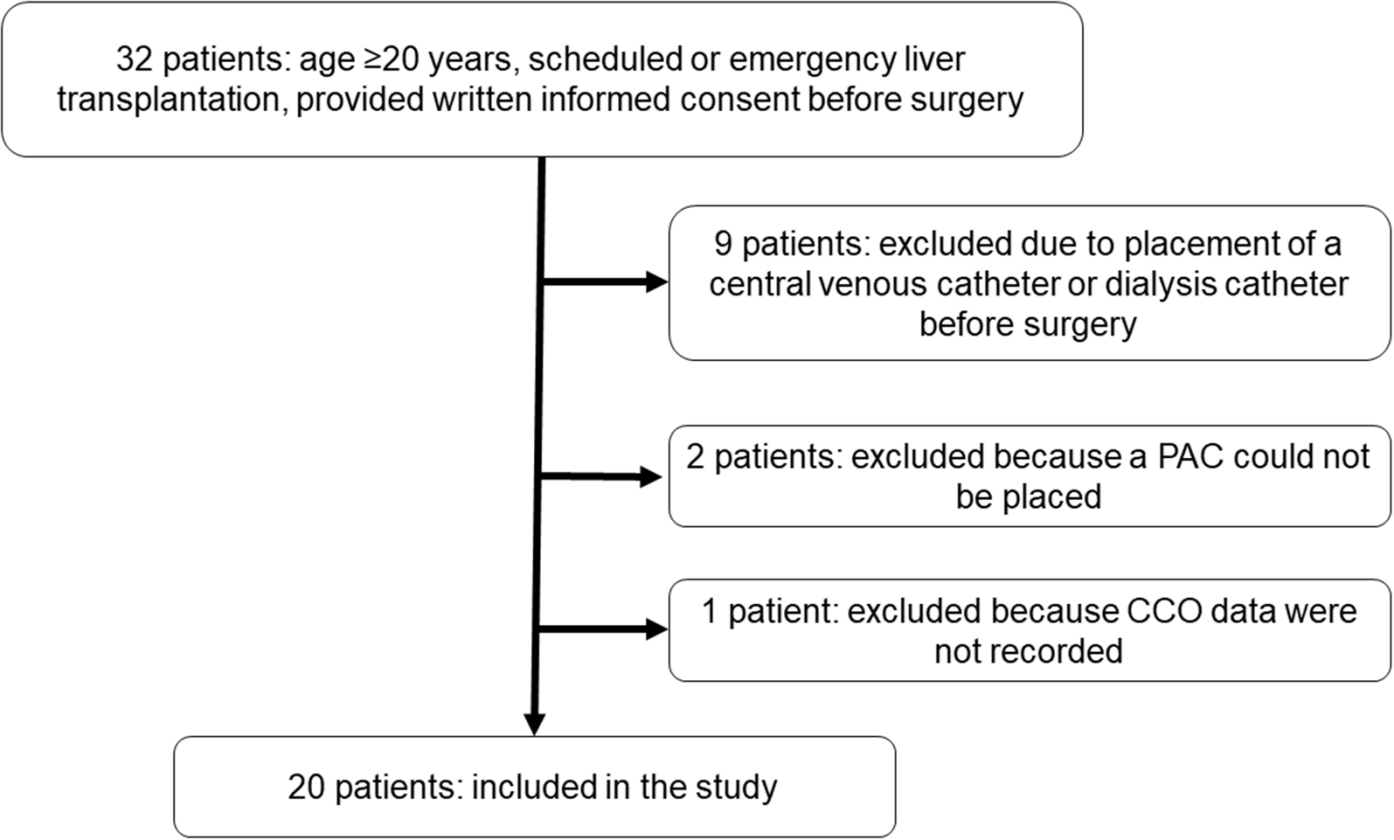
Flow diagram of inclusion and exclusion of patients in the study. A total of 32 patients aged ≥ 20 years old underwent scheduled or emergency liver transplantation. Twelve were excluded due to meeting the indicated exclusion criteria, and 20 were included in the study.

A total of 32 patients aged ≥ 20 years old underwent scheduled or emergency liver transplantation at our hospital in the study period (Fig. 1). All patients provided written informed consent before surgery. Twelve patients were excluded due to placement of a central venous catheter or dialysis catheter in the internal jugular vein before surgery (*n* = 9), failure to place a PAC (*n* = 2), and no data recorded (*n* = 1). Thus, 20 patients were ultimately included in the study (Fig. 1). The patient characteristics are shown in Table 1.

**Table 1:** Patient characteristics (*n* = 20).

Item	Value
Age (years)	55 [44–61]
Sex (male/female)	15/5
Body surface area (BSA) (m^2^)	1.72 [1.62–1.87]
Model for End-Stage Liver Disease (MELD) score	15 [12–19]
Model for End-Stage Liver Disease (MELD)-Na score	19 [15–21]
**Liver disease**	
Liver cirrhosis	9
Primary sclerosing cholangitis	4
Primary biliary cirrhosis	3
Graft dysfunction after liver transplantation	1
Ornithine transcarbamylase deficiency	1
Budd Chiari syndrome	1
Biliary atresia	1
Scheduled/emergency surgery	19/1
Operation time (min)	755 [682–874]
Dissection phase (min)	235 [200–279]
Anhepatic phase (min)	223 [160–252]
Neohepatic phase (min)	309 [293–418]
Estimated blood loss (mL)	5513 [4023–6913]
**Total dose of vasopressor or catecholamine**	
Dopamine (μg)	0[0–0]
Dobutamine (μg)	0 [0–0]
Norepinephrine (μg)	105 [0–1100]
Vasopressin (IU)	0 [0–0]
Phenylephrine (mg)	1.7 [0.6–3.2]

Data are expressed as a number or median [25th-75th percentile].

**Figure 2:**
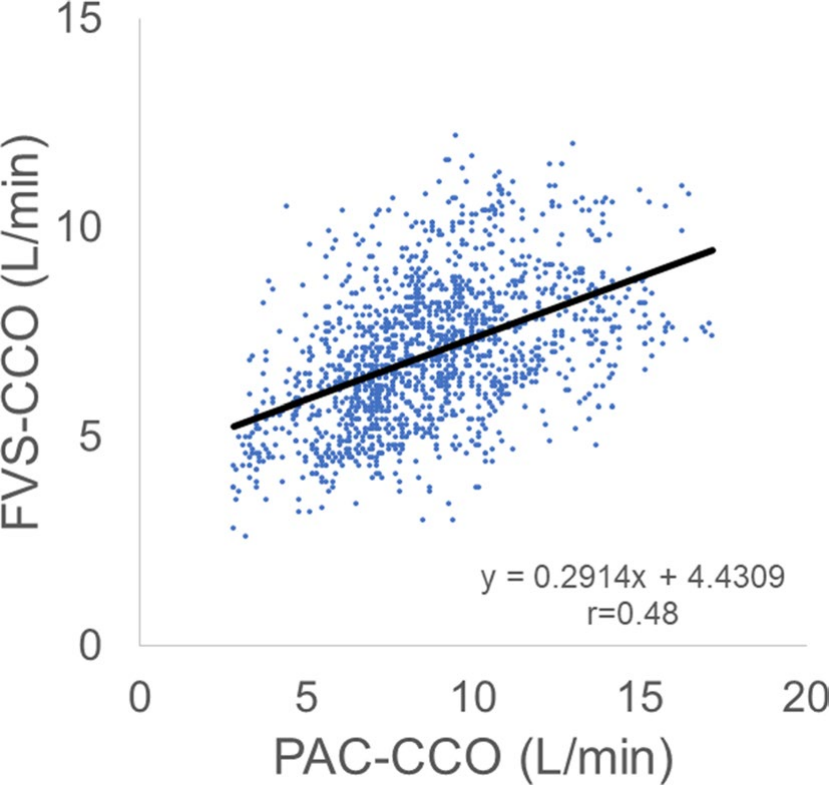
Linear regression and Pearson correlation analysis for all data pairs. The Pearson correlation coefficient (r) for PAC-CCO vs. FVS-CCO was 0.48, indicating a moderate correlation.

**Figure 3:**
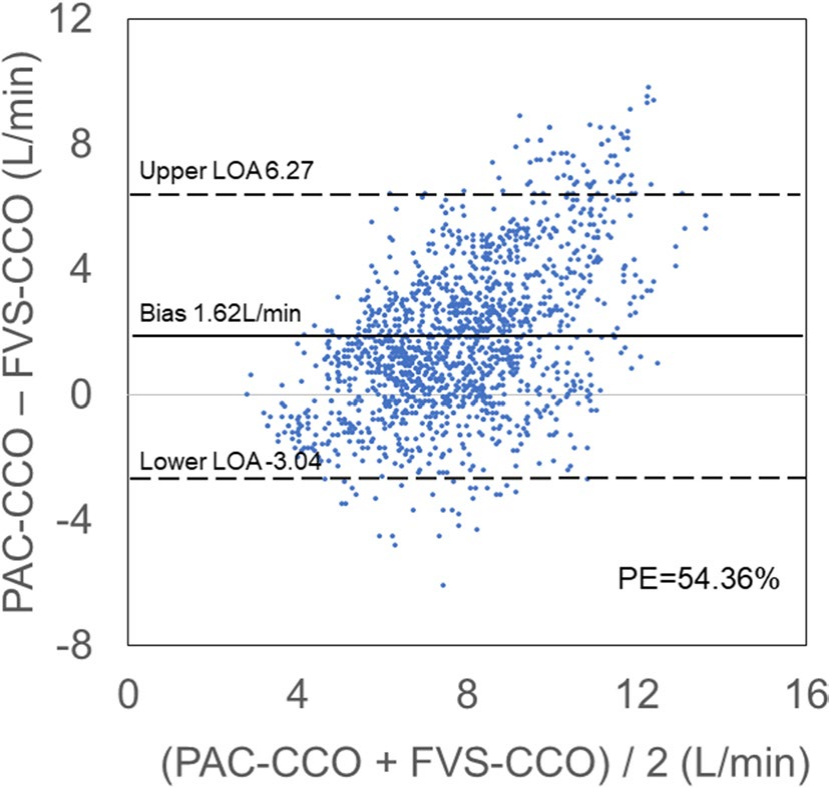
Bland–Altman plot for all data pairs. In Bland–Altman analysis, bias was 1.62 L/min, the 95% limits of agreement (LOA) were -3.04 to 6.27, and the percentage error (PE) was 54.36%.

A total of 1517 PAC-CCO vs. FVS-CCO data pairs were obtained. These data were evaluated using Pearson correlation coefficients (*r*) (Fig. 2), linear regression analysis (Fig. 3), and Bland–Altman plots and analysis (Table 2). In all data pairs, the mean PAC-CCO was 8.73 L/min and the mean systemic vascular resistance (SVR) was 617.5 dyne·s·cm^-5^. The *r* for PAC-CCO vs. FVS-CCO was 0.48, showing a moderate correlation (Fig. 2), however a correlation coefficient measures the strength of a relationship between two variables, not the agreement between them. In Bland–Altman analysis, the bias was 1.62 L/min, the 95% limits of agreement (LOA) were  − 3.04 to 6.27, and the percentage error (PE) was 54.36% (Fig. 3). In data pairs with SVR ≥ 800 dyne·s·cm^-5^, the bias was close to 0, but the PE was high.

**Table 2:** Results of Bland–Altman analysis and Pearson correlation analysis.

	*N* (data pairs)	mean PAC-CCO (l/min)	mean SVR (dyne·s· cm^-5^)	Bias (L/min)	SD (L/min)	95% limits of agreement	Percentage error (%)	*r*
All data pairs	1517	8.73	617.5	1.62	2.37	-3.04 to 6.27	54.36	0.48
SVR < 800 dyne·s·cm^-5^	1238	9.44	505.95	1.86	2.22	-2.49 to 6.21	47.03	0.41
SVR ≥ 800 dyne·s·cm^-5^	272	5.59	1125.18	0.27	2.05	-3.76 to 4.29	73.38	0.44
Dissection phase	425	7.88	707.4	0.82	2.19	-3.47 to 5.10	55.5	0.49
Anhepatic phase	429	8.18	663.43	1.52	2.33	-3.04 to 6.08	56.89	0.48
Neohepatic phase	663	9.64	529.89	2.19	2.26	-2.24 to 6.62	46.94	0.46

**Figure 4:**
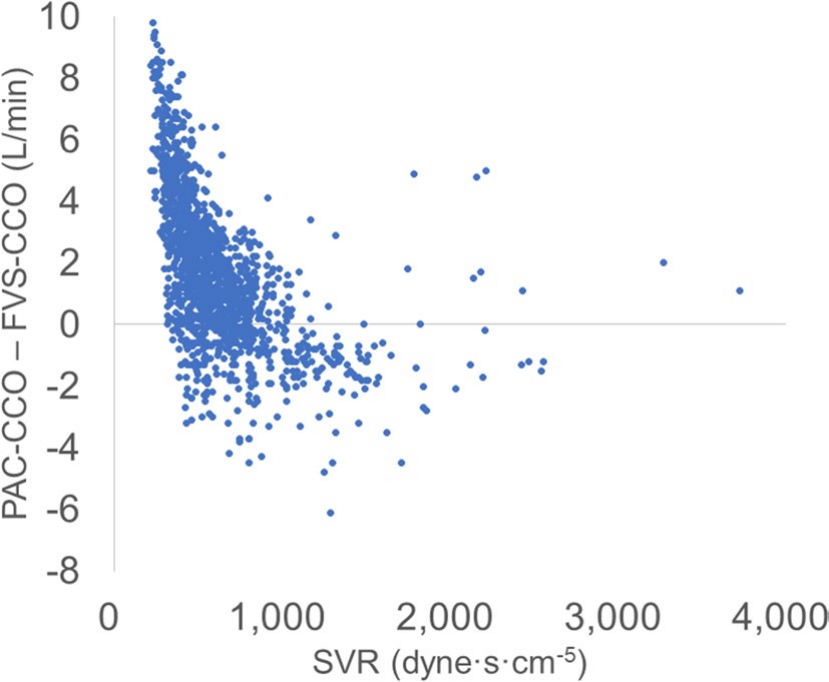
Plot of SVR against the differences between PAC-CCO and FVS-CCO. Seven SVRs were missing (*n* = 1510). Very large difference was shown in the low SVR region.

**Figure 5:**
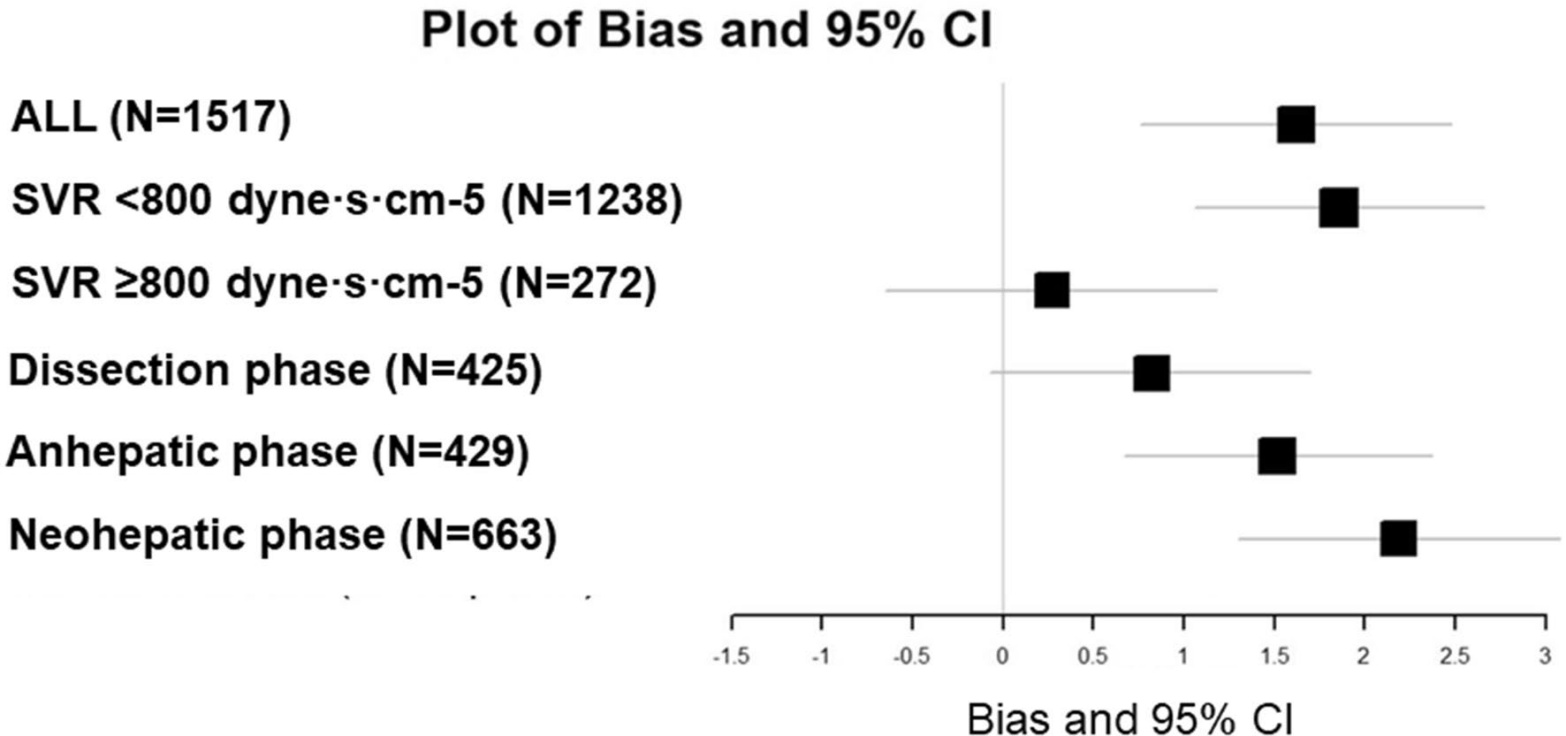
Plots of bias and 95% confidence interval (95% CI) for all data pairs, in data pairs with SVR < 800 and ≥ 800 dyne·s·cm^-5^, and in each surgical phase. Bias was close to 0 in cases with SVR ≥ 800 dyne·s·cm^-5^ and in the dissection phase.

**Figure 6:**
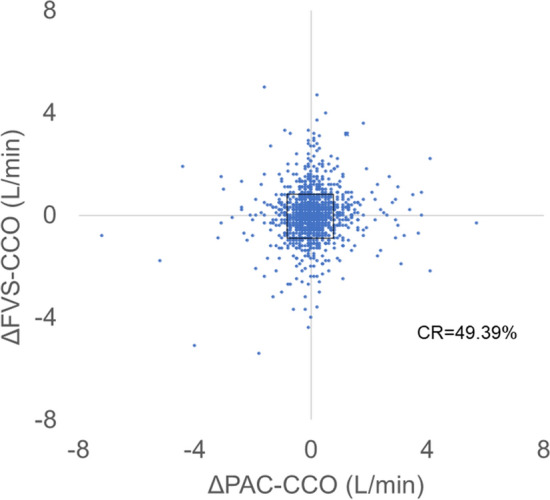
Four-quadrant plot for all data. The concordance rate (CR) of all data pairs was 49.39%. The exclusion zone was 0.873 L/min, which is 10% of the mean PCO.

In the dissection phase, the mean PAC-CCO and SVR were relatively normal and the bias was low, but tended to increase as the surgical phase progressed. Plot of SVR against the differences between PAC-CCO and FVS-CCO showed very large difference in the low SVR region (Fig. 4). Bias and 95% confidence interval (95% CI) plots for each group showed that bias was close to 0 in data pairs with SVR ≥ 800 dyne·s·cm^-5^ and in the dissection phase (Fig. 5). In the 4-quadrant plot (Fig. 6), the concordance rate (CR) was about 50% for all data pairs or any classification (Table 3).

**Table 3:** Results of 4-quadrant plot analysis.

Item	*N* for trending analysis	Concordance rate (%)
All data pairs	494	49.39
SVR < 800 dyne·s·cm^-5^	405	50.37
SVR ≥ 800 dyne·s·cm^-5^	87	44.83
Dissection phase	143	50.35
Anhepatic phase	178	44.94
Neohepatic phase	173	53.18

Pearson correlation coefficients and patient characteristics are shown in Table 4. Data for each patient are shown in Supplemental Tables 1 and 2. Body surface area (BSA) had a moderate positive correlation with mean PAC-CCO and a moderate negative correlation with mean SVR. The Model for End-Stage Liver Disease (MELD) and MELD-Na scores had moderate negative correlations with mean SVR and weak-moderate negative correlations with PE and CR. MELD-Na scores had a slightly stronger negative correlation with mean SVR, PE and CR, compared to MELD scores. Mean SVR had a strong negative correlation with mean PAC-CCO and bias, and a moderate positive correlation with PE.

**Table 4:** Pearson correlation coefficients and patient profile data.

Parameter	mean PAC-CCO (l/min)	mean SVR (dyne·s· cm^-5^)	Bias (L/min)	*r* (PAC-CCO vs. FVS-CCO)	Percentage error (%)	Concordance rate (%)
BSA	0.47	− 0.51	0.39	− 0.02	− 0.32	− 0.26
MELD score	0.32	− 0.46	0.33	− 0.20	− 0.21	− 0.30
MELD-Na score	0.34	− 0.52	0.35	− 0.05	− 0.25	− 0.40
Mean SVR	− 0.89	−	− 0.69	0.05	0.57	0.18

Data in this table were calculated based on the individual patient data in Supplemental Tables 1 and 2.

## Discussion

In this study, we showed that agreement of fourth-generation FVS-CCO with PAC-CCO in adult liver transplant recipients with end-stage liver failure was poor in bias, PE, and CR of 4-quadrant plots, and did not meet predetermined criteria^6,7^. Previous studies have found bias of third-generation FVS and PAC-ICO of 0.4–1.17 L/min, PE of 37.50–64.40%, and CR of 63.0–74.0% in patients with end-stage liver failure^4,8–10^. Studies in cardiac surgery have reported bias of fourth-generation FVS and PAC-CCO of -0.69–0.09 L/min, PE of 51.80–69.70%, and CR of 50.9–64.1%^11,12^. Our results are consistent with these studies, indicating no improvement in accuracy of the fourth-generation FVS compared to the third-generation FVS in adult liver transplantation.

Our results showed particularly high bias in the low SVR region. The third-generation FVS (ver. 3.02) had slightly improved bias against PAC-ICO in low SVR cases, compared to the second-generation FVS (ver. 1.10), but PE and CR did not improve sufficiently to meet the Critchley criteria^4,9^. In addition, as surgery progressed, the hyperdynamic state and the bias increased. Liver transplantation has three distinct stages: the dissection, anhepatic, and neohepatic phases, and each stage has its own hemodynamic concerns^1^. The dissection phase is characterized by massive blood loss and intermittent compression of the inferior vena cava due to surgical procedures; the anhepatic phase by cross-clamping of the portal vein and inferior vena cava, which reduces preload and reduces CO by up to 50%; and the neohepatic phase by hemodynamic instability due to donor liver reperfusion with release of inflammatory and vasodilator mediators. Each surgical step may increase hemodynamic instability.

FVS-CCO is calculated using an algorithm that includes pulse rate, standard deviation of the arterial pressure, and an auto-calibration factor χ calculated from the waveform. Unlike the third-generation FVS algorithm, which calculates χ every minute, the fourth-generation algorithm adds a new component (K fast), which is updated every 20 s, to χ. This may allow the fourth-generation FVS to compensate for rapid changes in vascular compliance due to administration of vasopressors and improve the trending ability against PAC-ICO^5^. However, this limited improvement did not lead to improvement in trending ability throughout surgery, and our study showed low trending ability, similar to previous studies of cardiac surgery or the third-generation FVS. Our findings show that data pairs with SVR ≥ 800 dyne·s·cm^-5^ and the dissection phase are associated with small bias.

There are several differences between this study and previous studies comparing FVS and PAC CO measurements in liver transplantation. ​First, we used PAC-CCO values as a control. In liver transplantation, anesthesiologists measure CO frequently due to hemodynamic instability^1,13^, and PAC-ICO is not suitable for frequent monitoring, although it has been used as a control in some studies. Moreover, the accuracy of PAC-ICO depends on user-dependent techniques such as the speed, volume and temperature of the injectate, as well as timing with respect to the respiratory cycle^1^. A meta-analysis reports the interchangeability between PAC-ICO and PAC-CCO, they found bias between PAC-ICO and PAC-CCO of 0.08 (95% CI 0.01 to 0.16) L/min, a PE of 29.7%, and bias in liver transplantation of 0.07 (95% CI − 0.26 to 0.40) L/min^2^. In low SVR cases, PAC-CCO has smaller differences with PAC-ICO than FVS-CCO^9^. Therefore, recent studies comparing FVS and PAC CO have used PAC-CCO as a control to assess FVS-CCO^11,12^.

A second difference is that we did not use averaged FVS-CCO values. FVS values update every 20 s, which is a shorter interval than that for PAC measurements. PAC-CCO takes at least 6 min to update and PAC-ICO takes > 10 min to average several measurements. Some studies have used average FVS-CCO values over several minutes^4,8,11,13^, whereas others do not use averages^9,10,12^. Thus, it is difficult to compare CO values measured at the same time, even with averaging^11^, and the most important clinical point is that anesthesiologists should not evaluate or make a decision based on the "average FVS-CCO". Third, we assessed all data points during the operation, without predefining these time points. In previous studies, the data collection points were predetermined based on the operative procedure^1,8,9,12,13^ or in the hemodynamically stable period^11,14^, which may not be a good reflection of clinical reality.

The MELD score is a predictor of survival in liver cirrhosis and has been adopted by the United Network for Organ Sharing (UNOS) as a criterion for transplant indication. The mean MELD score of 18 in the current study is lower than those of 21–24 in several previous liver transplantation studies^4,8,13^, although one study found a mean MELD score of 11^9^. The MELD-Na score includes serum Na levels in the calculation and is a more accurate predictor of survival^15^. Cases with a high MELD score have a significantly lower SVR^16^ and MELD-Na scores have a stronger negative correlation with SVR, compared to MELD scores^17^. In the current study, the MELD and MELD-Na scores were also negatively correlated with mean SVR, and MELD-Na scores showed a stronger negative correlation with PE and CR, compared to MELD scores. There is currently no method to predict the accuracy of FVS-CCO for each patient preoperatively. Our results suggest that the MELD-Na score may be an indicator of hemodynamic stability and FVS-CCO reliability in liver transplantation for patients with end-stage liver failure.

### Limitations

This study has several limitations. First, this study was a single-center study, which may have introduced bias in surgical techniques and patient types. Besides, we had a reduced number of target cases due to a decrease in the number of operations causes by the COVID-19 pandemic. However, we consider this to be an appropriate number of patients, as the sample sizes of previous studies have been around 20–30^4,8–13^. Second, anesthesia management was not standardized, including use of vasopressors or catecholamines. However, uniform management is difficult in liver transplantation due to the hemodynamic instability during the entire surgical period.

## Conclusion

Agreement and trending between fourth-generation FVS-CCO and PAC-CCO were found to be low in adult liver transplant recipients. Thus, we do not recommend use of FVS-CCO in adult liver transplantation.

### Methods

The study was approved by the ethics committee of Kyoto University Hospital (R2089) and carried out according to the guidelines of the Declaration of Helsinki. All methods were performed in accordance with the institutional guidelines and regulations.

#### Patients and anesthesia

All patients aged ≥ 20 years old who underwent scheduled or emergency liver transplantation at Kyoto University Hospital from September 2019 to June 2021 were eligible for the study. All patients provided written informed consent before surgery. The exclusion criteria were patients in whom a PAC could not be placed, accurate and regular arterial pressure waveforms could not be obtained, or a data recording failure occurred. After general anesthesia was introduced, the patient was intubated and mechanically ventilated. A central venous catheter (CVC) and PAC (Swan-Ganz pulmonary artery catheter with continuous thermodilution, Edwards Lifesciences, Irvine, CA) were inserted into the right internal jugular vein under echo guidance, and the PAC was placed in the main branch of the pulmonary artery under waveform guidance. The correct position of the PAC was confirmed by X-ray before the start of surgery. The inserted PAC was connected to a hemodynamic monitoring system vigilance II monitor (Edwards Lifesciences) and calibrated based on blood gas measurements. We did not recalibrate PAC-CCO and measure PAC-ICO. SVRs were automatically calculated by the formula: SVR = 80 × (Mean Arterial Pressure—Mean Venous Pressure or CVP) / CO. A 22G vascular catheter (BD Insyte 22G, Becton, Dickinson and Company, Franklin Lakes, NJ) was inserted into the radial artery and connected to the fourth-generation FloTrac/Vigileo system (ver. 4.00, Edwards Lifesciences). Patient data (age, sex, body weight, and height) were entered in the FVS and the system was zeroed at the mid-axillary line before CO measurements were initiated. CCO measured simultaneously by the two methods and SVR were recorded every minute from the start of measurement to the end of surgery. Values measured by the FVS sensor were hidden from the anesthesiologist in charge throughout the entire study period. The patency of the arterial line was confirmed by the anesthesiologist in charge every hour. Circulatory management during surgery was guided by PAC readings and followed standard practice at our hospital. In case of hypotension, vasoconstrictors and catecholamines (noradrenaline, dopamine, and dobutamine) were used at the discretion of the anesthesiologist in charge. Liver transplantation was performed by a single surgical team. All patients were transferred to the intensive care unit after surgery.

#### Data collection

Age, sex, BSA, liver disease, and blood test data were collected from electronic medical records. MELD and MELD-Na scores were calculated from the blood test data immediately before surgery as: MELD score = 10 × ((0.957 × ln(Creatinine)) + (0.378 × ln(Bilirubin)) + (1.12 × ln(INR)) + 6.43; MELD-Na score = MELD score − Serum Na − (0.025 × MELD score × (140 − Serum Na)) + 140"^15^. Operation time, duration of each operation phase, estimated blood loss, use of vasopressors or catecholamines, CO by both measurement methods, and SVR values were obtained from anesthesia record data. The surgical phases were defined as follows: the dissection phase from the start of anesthesia or both CCO measurements to hepatic vein amputation or hepatic vein resection; the anhepatic phase from hepatic vein dissection or hepatic vein resection to hepatic vein reperfusion; and the neohepatic phase from hepatic vein reperfusion to the end of both CCO measurements or the end of surgery. The PAC-CCO system provides CO values as a mean of the last 6 thermodilution measurements, with a maximum of 9 min needed to estimate the CO value, as a single measurement takes 1–1.5 min^11^. The fourth-generation FVS provides an update every 20 s^11^. Therefore, we extracted a set of data every 10 min in order to avoid overlapping of values.

#### Data analysis

Using the extracted data, statistical analysis was performed for all data pairs, data pairs with SVR < 800 and ≥ 800 dyne·s·cm^-5^, in each surgical phase, and in each case. Pearson correlation coefficient (*r*) analysis between two variables, linear regression with Bland–Altman analysis adjusted for repeated measures^14,18^, and 4-quadrant plot analysis were performed. Correlations between measured data and patient preoperative data were also evaluated by Pearson correlation analysis. Correlations were defined as zero (r = 0.0), weak (r = 0.1–0.3), moderate (r = 0.4–0.6), strong (r = 0.7–0.9), and perfect (r = 1.0)^19^. Bias is the mean difference of two measurements. Limits of agreement (LOA) is mean difference ± 1.96 × standard deviation of difference. Percentage error is 2 × standard deviation of difference/mean measurements of the reference methods. The exclusion zone of the 4-quadrant plot was set to 0.873 L/min, which is 10% of the mean PCO. The evaluation criterion for the degree of agreement in the Bland–Altman analysis was PE < 30%^7^. The criterion for concordance in 4-quadrant plot analysis was CR > 90% with an exclusion range of 10%^20^. R (R ver. 3.6.3, R Foundation for Statistical Computing, Vienna, Austria) and Excel 2016 (ver. 2111, Microsoft, Redmond, WA, US) were used for statistical analysis.

## Supplementary Information

Supplementary Information.

## Data Availability

The datasets generated and/or analyzed during the current study are available from the corresponding author on reasonable request.
