# Supplementary material for: Agreement between continuous cardiac output measured by the fourth-generation FloTrac/Vigileo system and a pulmonary artery catheter in adult liver transplantation
Source: Sci Rep. 2022 Jul 1;12:11198. doi: 10.1038/s41598-022-14988-z (PMC9249899; doi:10.1038/s41598-022-14988-z)
Supplement: Supplementary file 1 — Supplementary Information. [file 41598_2022_14988_MOESM1_ESM.docx]

**Supplemental Table 1.** Characteristics of each patient.

| Case Number | N  (Data pairs) | MELD score | MELD-Na score | Mean PAC-CCO　(l/min) | Mean SVR  (dyne·s· cm^-5^) | Total dose of　DOA (μg) | Total dose of DOB (μg) | Total dose of NAD (μg) | Total dose of vasopressin (U) | Total dose of phenylephrine (mg) |
| --- | --- | --- | --- | --- | --- | --- | --- | --- | --- | --- |
| Case 1 | 84 | 10 | 11 | 8.95 | 651.52 | 0 | 0 | 0 | 0 | 0.3 |
| Case 2 | 66 | 14 | 17 | 8.86 | 515.67 | 31992 | 0 | 61 | 0 | 10.2 |
| Case 3 | 109 | 15 | 17 | 12.38 | 504.84 | 0 | 0 | 0 | 0 | 3.9 |
| Case 4 | 78 | 25 | 29 | 8.89 | 474.66 | 0 | 0 | 3020 | 0 | 0.0 |
| Case 5 | 69 | 19 | 21 | 6.49 | 774.25 | 127950 | 0 | 2542 | 0 | 2.4 |
| Case 6 | 84 | 8 | 10 | 5.16 | 1023.16 | 0 | 0 | 160 | 0 | 0.3 |
| Case 7 | 61 | 12 | 22 | 10.41 | 410.77 | 80850 | 80850 | 620 | 5 | 5.1 |
| Case 8 | 84 | 14 | 19 | 6.18 | 818.50 | 0 | 0 | 500 | 0 | 0.9 |
| Case 9 | 45 | 26 | 27 | 8.15 | 587.98 | 0 | 0 | 182 | 0 | 2.4 |
| Case 10 | 55 | 11 | 14 | 5.75 | 771.55 | 0 | 0 | 2542 | 0 | 1.2 |
| Case 11 | 70 | 15 | 16 | 9.57 | 483.64 | 0 | 0 | 0 | 0 | 1.9 |
| Case 12 | 58 | 20 | 20 | 10.40 | 496.98 | 0 | 0 | 0 | 0 | 0.6 |
| Case 13 | 119 | 21 | 21 | 12.63 | 355.67 | 0 | 0 | 5337 | 0 | 0.5 |
| Case 14 | 55 | 13 | 13 | 8.35 | 582.78 | 0 | 0 | 0 | 0 | 1.6 |
| Case 15 | 69 | 22 | 23 | 8.71 | 548.55 | 0 | 0 | 150 | 0 | 0.0 |
| Case 16 | 88 | 12 | 19 | 10.99 | 410.69 | 0 | 0 | 0 | 0 | 8.5 |
| Case 17 | 123 | 17 | 19 | 7.37 | 755.52 | 104610 | 0 | 3970 | 24 | 9.6 |
| Case 18 | 66 | 8 | 8 | 5.71 | 991.89 | 0 | 0 | 0 | 0 | 2.0 |
| Case 19 | 50 | 15 | 15 | 9.26 | 432.54 | 0 | 0 | 0 | 0 | 3.0 |
| Case 20 | 84 | 14 | 18 | 7.14 | 752.93 | 0 | 0 | 0 | 0 | 1.6 |

**Supplemental Table 2.** Results of analysis for each patient.

| Case Number | Bias (L/min) | SD of Bias (L/min) | 95% Limits of Agreement | Percentage Error (%) | *r* | N for trending analysis | Concordance rate (%) |
| --- | --- | --- | --- | --- | --- | --- | --- |
| Case 1 | 2.62 | 0.89 | 0.87 to 4.37 | 19.95 | 0.81 | 19 | 78.95 |
| Case 2 | 1.65 | 1.37 | -1.04 to 4.34 | 31.02 | 0.23 | 30 | 50 |
| Case 3 | 4.5 | 2.46 | -0.32 to 9.33 | 39.79 | 0.4 | 53 | 52.83 |
| Case 4 | 0.57 | 1.74 | -2.84 to 3.98 | 39.18 | 0.26 | 17 | 47.06 |
| Case 5 | 1.32 | 1.11 | -0.86 to 3.50 | 34.3 | 0.57 | 24 | 41.67 |
| Case 6 | -0.43 | 1.03 | -2.46 to 1.59 | 40.01 | 0.72 | 17 | 64.71 |
| Case 7 | 2.54 | 1.38 | -0.15 to 5.24 | 26.42 | 0.76 | 35 | 37.14 |
| Case 8 | -0.65 | 1.16 | -2.94 to 1.63 | 37.68 | 0.64 | 22 | 59.09 |
| Case 9 | 2.73 | 0.95 | 0.86 to 4.60 | 23.42 | 0.5 | 11 | 36.36 |
| Case 10 | -0.5 | 1.38 | -3.20 to 2.21 | 48.01 | 0.31 | 17 | 41.18 |
| Case 11 | -0.09 | 1.29 | -2.62 to 2.44 | 26.97 | 0.67 | 32 | 59.38 |
| Case 12 | 3.8 | 1.41 | 1.04 to 6.55 | 27.05 | 0.59 | 13 | 38.46 |
| Case 13 | 4.86 | 2.38 | 0.19 to 9.54 | 37.75 | 0.19 | 39 | 43.59 |
| Case 14 | 0.13 | 0.87 | -1.58 to 1.84 | 20.84 | 0.38 | 20 | 40 |
| Case 15 | 1.97 | 0.96 | 0.09 to 3.85 | 22 | 0.72 | 25 | 56 |
| Case 16 | 4.84 | 0.81 | 3.26 to 6.42 | 14.66 | 0.66 | 17 | 41.18 |
| Case 17 | 0.81 | 1.35 | -1.83 to 3.44 | 36.5 | 0.36 | 31 | 51.61 |
| Case 18 | -0.55 | 1.55 | -3.59 to 2.49 | 54.32 | 0.3 | 19 | 42.11 |
| Case 19 | 1.7 | 1.51 | -1.25 to 4.65 | 32.52 | 0.38 | 20 | 60 |
| Case 20 | 0.5 | 1.78 | -2.99 to 4.00 | 49.93 | 0.32 | 33 | 42.42 |
